# Fibroblast Growth Factor 21 and Browning of White Adipose Tissue

**DOI:** 10.3389/fphys.2019.00037

**Published:** 2019-02-05

**Authors:** Daniel Cuevas-Ramos, R. Mehta, Carlos A. Aguilar-Salinas

**Affiliations:** ^1^Department of Endocrinology and Metabolism, Instituto Nacional de Ciencias Médicas y Nutrición Salvador Zubirán, Mexico City, Mexico; ^2^Unidad de Investigación de Enfermedades Metabólicas, Instituto Nacional de Ciencias Médicas y Nutrición Salvador Zubirán, Mexico City, Mexico; ^3^Instituto Tecnológico y de Estudios Superiores de Monterrey Tec Salud, Monterrey, Mexico

**Keywords:** fibroblast growth factor 21, glucose, energy balance, insulin resistance, irisin, exercise, noradrenaline, free fatty acids

## Abstract

Interest has been focused on differentiating anatomical, molecular, and physiological characteristics of the types of mammalian adipose tissues. White adipose tissue (WAT) and brown adipose tissue (BAT) are the two main forms of adipose tissue in humans. WAT functions as an endocrine organ and serves as a reservoir of energy in the form of triglycerides. The hormones released by WAT are called adipokines. BAT consists of a group of specialized cells with abundant uncoupling protein 1 (UCP1) in the inner mitochondrial membrane and also fulfills endocrine functions. Following the identification of functional (BAT) in human adults, there has been a great deal of interest in finding out how it is induced, its localization, and the mechanisms by which it regulates thermogenesis. Fibroblast growth factor 21 (FGF21) is a key regulator of the differentiation to brown adipocytes. The main mechanisms occur through enhancing UCP1 expression. In addition, following exposure to cold or exercise, FGF21 induces upregulation of local peroxisome proliferator-activated receptor gamma co-activator (PGC)-1-alfa and thus promotes thermogenesis in adipose tissue and skeletal muscle. FGF21 integrates several pathways allowing the regulation of human energy balance, glucose levels, and lipid metabolism. Such mechanisms and their clinical relevance are summarized in this review.

## Introduction

Fibroblast growth factor 21 (FGF21) has important effects on energy balance, glucose metabolism, and lipid metabolism (Kharitonenkov et al., 2005). Initial reports identified the liver as its main source (Nishimura et al., 2000). On secretion, its most important target is white adipose tissue (WAT), where FGF21 increases expression of GLUT1 and consequently glucose uptake (Kharitonenkov et al., 2005). During fasting or starvation, lipolysis is triggered, with a subsequent increment in circulating free fatty acids (FFAs). FFAs induce the activation of the peroxisome proliferator-activated receptor (PPAR)-alfa in the liver, resulting in the synthesis and release of FGF21. Since carbohydrate ingestion is absent during starvation, FGF21 induces ketone body formation in the liver as an additional energy source (Badman et al., 2007; Inagaki et al., 2008). FGF21 may also be considered an adipokine, since it is also synthesized and released from WAT. It shows complementary actions to adiponectin (Lin et al., 2013), increasing insulin sensitivity, improving the lipid profile, reducing glucose levels without causing hypoglycemia, and ensuring energy availability during starvation (Badman et al., 2007; Inagaki et al., 2008). In human insulin resistance states, FGF21 levels have a positive correlation with the number of metabolic syndrome traits, the severity of oxidative stress, and the presence of type 2 diabetes (Zhang et al., 2008; Cuevas-Ramos et al., 2010; Gómez-Sámano et al., 2017). Other human stress-related conditions that increase circulating FGF21 levels are lactation (Schoenberg et al., 2011), exercise (Cuevas-Ramos et al., 2012b), growth hormone treatment (Chen et al., 2011), and anorexia nervosa (Dostálová, 2008). Interestingly, FGF21 is now considered an important mediator for decreasing oxidative stress, and possibly, preventing microvascular diseases such as diabetic nephropathy (Jian et al., 2012).

The role of FGF21 as a therapeutic option in human metabolic diseases is of increasing importance. Currently, there are multiple recombinant FGF21 analogs in phase 2 and phase 3 clinical trials (Mu et al., 2012; Gaich et al., 2013; Talukdar et al., 2016). Interest has increased even more so after the discovery of the crucial role of FGF21 in inducing the proliferation of brown adipose tissue (BAT) (Fisher et al., 2012). This review describes the mechanisms by which FGF21 induces “browning” of adipose tissue and how it may have a role in the treatment of human metabolic diseases, including obesity and type 2 diabetes.

## White, Beige, and Brown Subtypes of Adipose Tissues in Humans

WAT and BAT are the two main subtypes of adipose tissue in humans. WAT has important endocrine functions in addition to its role as a reservoir of energy in the form of triglycerides. The hormones released from WAT, namely adipokines, are a well-recognized group of bioactive factors with endocrine actions that act through specific cell-membrane receptors. Adipokines trigger certain intracellular signaling pathways, which modulate human metabolism (Piya et al., 2013). The most important are leptin and adiponectin, but visfatin, chemerin, omentin, hepcidin, apelin, and vaspin have also been described (Piya et al., 2013).

BAT is also an important endocrine organ. It consists of a group of specialized cells with abundant expression of uncoupling protein 1 (UCP1) in the inner mitochondrial membrane (Aherne and Hull, 1966; Cannon and Nedergaard, 2004). The BAT hormones are named “batokines” (Baboota et al., 2015; Booth et al., 2016). The principle function of BAT is to dissipate stored energy in the form of heat by uncoupling energy oxidation from ATP synthesis (Fedorenko et al., 2012). Initially, BAT was only considered as an energy-producing organ in rodents and human infants (Bartness et al., 2010). However, after the development of 18F-fluorodeoxyglucose (FDG) positron emission tomography-computed tomography (PET-CT), BAT has also been identified in human adults (Nedergaard et al., 2007; Cypess et al., 2009). Nevertheless, the origin of BAT is still under debate, although originally it was thought to be derived from skeletal muscle-like lineage (Myf5+) (Seale et al., 2007). In human adults, a type of adipose tissue showing characteristics between that of white and brown adipocytes has been identified; this kind of adipose tissue is known as beige adipose tissue (*brite*, brown-in-white) (Jespersen et al., 2013). It appears that beige and white adipocytes arise from both Myf5+ and Myf5- progenitor cells. These findings confirm that the skeletal muscle-like lineage is not the only source of BAT (Wu et al., 2012).

The location of the different types of adipose tissue varies. Beige and WAT have mainly visceral (mesenteric, perigonadal or omental adipose tissue surrounding organs) and subcutaneous (under skin) locations. BAT is located only in axillary, subscapular, interscapular, and periaortic regions in rodents; in cervical, supraclavicular, paravertebral, mediastinal regions in humans; and in perirenal regions in both (Park et al., 2014; Sanchez-Gurmaches and Guertin, 2014). *Brite* or beige adipocytes have basal metabolic actions similar to those seen in white adipocytes, and with the enough stimulus, they are able to transform into thermogenic adipocytes with higher UCP1 expression similar to BAT (Wu et al., 2012). This process is referred as “browning” and it describes the capacity of white adipocytes to acquire a phenotype similar to that of BAT, leading to increased thermogenesis. It is achieved when white adipocytes are exposed to cold or to beta 3-adrenoreceptor agonists (Young et al., 1984, Harms and Seale, 2013). Browning occurs mainly in subcutaneous white adipose fat depots. The underlying molecular mechanisms for this trans-differentiation are currently under intensive research (Luo and Liu, 2016). In addition, there are important structural differences among WAT, *brite*, and BAT. WAT is a large lipid droplet, with a peripheral nucleus and a small amount of cytoplasm, whereas BAT has a central nucleus with more cytoplasm but smaller lipid droplets. In between these is the *brite* or beige tissue; this has the mixed structural characteristics of both. Sometimes the different structures are found together; for example, the ectopic expression of UCP1 and the presence of the PR domain containing 16 (PRDM16) suggests that brite adipocytes are mixed with white adipocyte depots (Wu et al., 2013). The balance between WAT and BAT, and their endocrine regulation, are key elements to better understand the development of weight gain and human metabolic diseases.

## Molecular Pathways and Clinical Relevance of Browning Induced by FGF21

Since the discovery of FGF21, it has been appreciated that its synthesis is strongly related to cold exposure (Badman et al., 2007; Inagaki et al., 2008). In mice, during hypothermia, FGF21 induces torpor, a short-term hibernation state in which animals can save energy by reducing body temperature and physical activity (Badman et al., 2007). More recently, studies have shown a higher expression of FGF21 in inguinal WAT after cold exposure. The role of FGF21 produced in WAT includes both paracrine and autocrine actions; this results in the local upregulation of peroxisome proliferator-activated receptor gamma co-activator (PGC)-1-alfa and thus an increase in thermogenesis (Hondares et al., 2010; Fisher et al., 2012; Adams et al., 2013; Emanuelli et al., 2014). PGC1-alfa is a protein involved in modulating several effects in post-exercise skeletal muscle, including the improvement of energy and glucose metabolism (Summermatter et al., 2013). Interestingly, PGC1-alfa is also induced after irisin or insulin exposure, both hormones showing a clear interaction with FGF21 post-exercise (Cuevas-Ramos et al., 2010, 2012b; Bostrom et al., 2012; Fisher et al., 2012; Hu and Christian, 2017). Irisin-induced phosphorylation of p38 mitogen-activated protein kinase (p38 MAPK) and extracellular signal-related kinase (ERK) show a positive correlation with shivering intensity (Bostrom et al., 2012; Zhang et al., 2014). FGF21 also shows a direct relationship with exercise intensity (Cuevas-Ramos et al., 2010, 2012b). The consequence of these PGC1-alfa inducers is to promote adaptive thermogenesis with “browning” of WAT (Fisher et al., 2012). The main mechanism following FGF21 action is PPAR-gamma activation in WAT, together with the irisin effect inducing MAPK and ERK pathways. This results in differentiation of pre-adipocytes to mature white adipocytes, which are then available for “browning” (Hondares et al., 2011; Zhang Y. et al., 2016).

**FIGURE 1:**
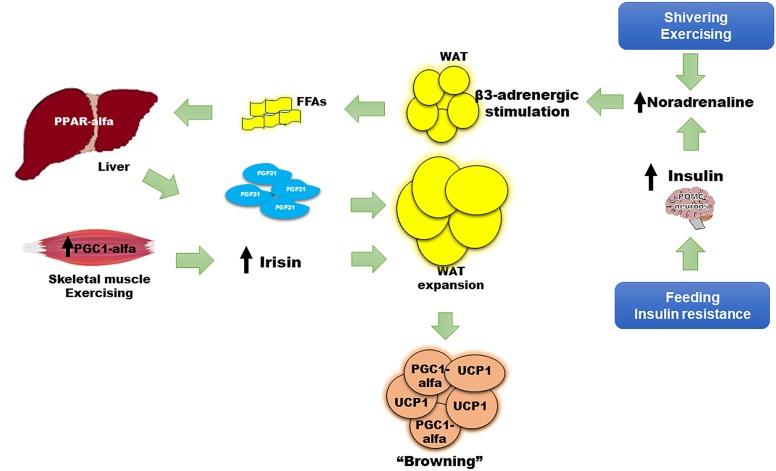
Role of FGF21 in the “browning” of adipose tissue. Adaptive thermogenesis following cold exposure, shivering, or exercise, and physiologic (i.e., feeding) or pathologic (i.e., insulin resistance) states, begins a compensatory process to induce “browning” of WAT, thus enhancing thermogenesis, energy waste, and improving cell metabolism. The principle mechanisms to induce “browning” involve insulin, irisin, and FGF21. Insulin increases adrenergic stimulation and noradrenaline secretion after acting on POMC neurons in the central nervous system. FGF21 also has a WAT-independent mechanism acting directly to the CNS, increasing noradrenaline release. Noradrenaline induces beta 3-adrenergic receptor stimulation and greater lipolysis that produces FFAs as main substrate for PPAR-alfa agonism and FGF21 synthesis and release from liver. Irisin is mainly released from skeletal muscle after shivering or exercise. Noradrenaline, irisin, and FGF21 promote uncoupling protein 1 (UCP1) expression, a protein that increases thermogenesis at the mitochondrial inner membrane, and upregulation of local peroxisome proliferator-activated receptor gamma co-activator (PGC)-1-alfa on white adipocytes, turning them to brite or brown adipose tissue. WAT expansion is also induced by FGF21, increasing insulin sensitivity.

Some animal models have reported findings consistent with these actions. For example, FGF21 deficiency in mice results in increased body weight with excessive adiposity, higher serum cholesterol, insulin resistance, and hyperglycemia (Kharitonenkov et al., 2005). The finding of a 30–40% lower nuclear content of PGC1-alfa at the hepatic mitochondrial level in *Fgf21* KO mice compared with WT mice, is a potential explanation for these results (Fletcher et al., 2016). FGF21 induces palmitate oxidation and β-hydroxyacyl-CoA dehydrogenase (β-HAD) activity. In the *Fgf21* KO model, these enzymatic activities are decreased, indicating lower lipid oxidation, a reduction in glucose metabolism, and a lower degree of energy waste (Fletcher et al., 2016). In contrast, overexpression of FGF21 effectively decreases weight, adiposity, levels of FFAs, triglycerides, glucose, and insulin, all due to the normalization of mitochondrial oxidation (Fletcher et al., 2016) and the improvement in insulin sensitivity (Kharitonenkov et al., 2005). Interestingly, exercise, irisin, and noradrenaline were necessary to restore PGC1-alfa content in the liver despite overexpression of FGF21, emphasizing the key interaction of such inducers with FGF21 (Fletcher et al., 2016). Insulin, the most important regulator of energy and glucose metabolism, enhanced differentiation of WAT to *brite* and brown adipocytes through pro-opiomelanocortin (POMC) neurons (Table 1 and Figure 1); (Dodd et al., 2015). Therefore, multiple mechanisms may be inter-connected to improve metabolism in humans, with FGF21 functioning as an important link between them.

**Table 1:** Most important hormones, drugs, and nutritional inducers of browning.

Effector	Effect and mechanism on WAT
AMPK activators	Higher thermogenesis, increase energy expenditure and mitochondrial biogenesis. Enhance PGC1-alfa and UCP1. Ej: with AICAR (Gaidhu et al., 2009)
BMPs	BMP7 and BMP 8 induce higher thermogenesis, increase energy expenditure and mitochondrial biogenesis. Enhance PGC1-alfa and UCP1. Increases lipid oxidation (Schulz et al., 2011; Whittle et al., 2012).
Beta-3-adrenergic stimulation	Higher thermogenesis, increase energy expenditure and mitochondrial biogenesis, enhance UCP1, and activation of c-AMP, PKA, p38 MAPK, PGC1-alfa, and PPAR-alfa (Jimenez et al., 2003; Li et al., 2005).
Fenofibrate	Effects are through PPAR-alfa agonism (Magliano et al., 2013; Rachid et al., 2015)
FGF21 recombinant analogs	After cold exposure or adrenergic stimulation induced higher thermogenesis, increase energy expenditure and mitochondrial biogenesis. Enhance PGC1-alfa and UCP1. After exercising, possible interaction with irisin reducing fat depots (Dostálová, 2008; Cuevas-Ramos et al., 2012b; Lee et al., 2014).
FGFR1/KLB antibodies	Higher thermogenesis but through UCP1-independent mechanism (Chen et al., 2017).
Insulin and leptin (adipoinsular axis)	Acts in hypothalamic POMC neurons to induce browning (Dodd et al., 2015).
Irisin	Higher thermogenesis, increase energy expenditure and mitochondrial biogenesis, enhance UCP1, through PPAR-alfa agonism. Irisin also stimulated browning after exercising (Bostrom et al., 2012; Wu et al., 2012; Zhang Y. et al., 2016).
Thyroid hormones	Higher thermogenesis, increase energy expenditure and mitochondrial biogenesis (Lee et al., 2012).
Natriuretic peptides (ANP)	Synergism with beta-3-adrenergic receptor stimulation after exercising inducing higher thermogenesis, enhancing UCP1 expression, and lipolysis through PKA, c-GMP and PKG (Lafontan et al., 2008; Bordicchia et al., 2012).
Thiazolidinediones	Higher thermogenesis, enhancing UCP1 expression and inducing insulin sensitivity. Synergism after adrenergic stimulus (Petrovic et al., 2010).
Capsaicin	Adrenergic stimulation causing higher thermogenesis, through TRPV1 protein activating neurons (Teodoro et al., 2014; Baskaran et al., 2016).
Bile acids	Higher thermogenesis, enhancing UCP1 expression through TGR5 (Watanabe et al., 2006; Teodoro et al., 2014).
Citrulline	Higher thermogenesis, increase energy expenditure and mitochondrial biogenesis, enhance UCP1, and PPAR-alfa agonism (Joffin et al., 2015).
Fucoxanthin	Higher thermogenesis, enhancing UCP1 expression (Maeda et al., 2005).
Luteolin	Higher thermogenesis, increase energy expenditure and mitochondrial biogenesis, enhance UCP1 (Zhang X. et al., 2016).
Methionine restriction	Higher thermogenesis, enhancing UCP1 expression (Hasek et al., 2010).
n-3 PUFAs	Higher thermogenesis, enhancing UCP1 expression (Zhao and Chen, 2014; Bargut et al., 2016).
Resveratrol	Higher thermogenesis, increase energy expenditure and mitochondrial biogenesis, enhancing PGC1-alfa and UCP1. Also increase PRDM16 expression, and increases lipid oxidation activating AMPK (Wang et al., 2015).
Retinoic acid	Higher thermogenesis, enhancing UCP1 expression. Also, PPAR-beta/delta expression (Murholm et al., 2013).
Beta-hydroxybutyrate	Higher thermogenesis, enhancing UCP1 expression (Carriere et al., 2014).

*AICAR, Activator 5-aminoimidazole-4-carboxamide ribonucleoside; AMPK, adenosin monophosphate activated protein kinase; ANP, atrial natriuretic peptide; c-GMP, cyclic-guanosine monophosphate protein; FFA, free fatty acids; FGF21, fibroblast growth factor 21; MAPK, Mitogen-activated protein kinase; PGC1-alfa, peroxisome proliferator-activated receptor gamma coactivator (PGC) 1-alfa; PKA, protein kinase A; PKG, protein kinase G; PPAR, peroxisome proliferator-activated receptor; PRDM16, PR domain containing 16; PUFA, polyunsaturated fatty acids; TRG5, G protein-coupled receptor 5; TRPV1, transient receptor potential V1; UCP1, uncoupled protein 1; WAT, white adipose tissue.*

The exogenous administration of an FGF21 analog in animal models has been associated with a thermogenic effect, an improvement in glucose homeostasis, lipid profile, and a reduction in body weight (Wu et al., 2011; Veniant et al., 2012). However, in humans, recombinant FGF21 analogs have not shown these effects. FGF21 is paradoxically increased in insulin-resistant states such as obesity or type 2 diabetes; this suggests either a resistance to FGF21 effects or a compensatory response to these metabolic disarrangements (Zhang et al., 2008; Cuevas-Ramos et al., 2010). Nevertheless, the key metabolic role of FGF21 is clear. Firstly, WAT increases FGF21 expression in response to feeding, which has an autocrine action on PPAR-gamma activity; PPAR-gamma is an important inducer of insulin sensitivity pathways, adipocyte maturation, and function (Dutchak et al., 2012). Secondly, FGF21 is significantly induced after moderate to intensive physical activity (Cuevas-Ramos et al., 2010, 2012b), and interestingly, its release has been correlated with sympathetic nervous system activation and lipolysis markers, such as noradrenaline and serum free fatty acids, respectively (Cuevas-Ramos et al., 2012b). Thirdly, FGF21 regulates metabolism through increasing insulin sensitivity at WAT and BAT, but this may happen in an acute and a chronic manner. Acute and chronic exogenous administration of FGF21 induce insulin sensitivity independently of adiponectin action (BonDurant et al., 2017). However, the chronic effect of FGF21 is not necessarily via a direct effect on WAT since it also induces signaling in the central nervous system via POMC neurons, and on brown adipocytes, enhancing thermogenesis and insulin-sensitivity in mice (Figure 1). These effects require functional insulin signaling in adipose tissue, otherwise the FGF21-benefit is lost. In addition, the action of FGF21 on the central nervous system is important to induce energy expenditure, mainly by increasing noradrenaline release (Figure 1) (Owen et al., 2014). Noradrenaline plays an important role in the regulation of “browning.” It is the most studied activator of thermogenesis, increasing UCP1 transcription, enhancing lipolysis and mitochondrial oxidation (Figure 1; (Puigserver et al., 1996). After cold-induced shivering or physical activity stress, noradrenergic pathways increase thermogenic gene expression through c-AMP-mediated mechanisms (Hondares et al., 2011). Lipolysis and FFAs increase following noradrenaline release. With acute intensive exercise, the increase in beta 3-receptor activity also causes an increment in circulating FGF21 (Cuevas-Ramos et al., 2012b). Increased circulating FFAs induce PPAR-alfa expression in the liver, increasing FGF21 synthesis and release into circulation (Badman et al., 2007; Inagaki et al., 2008). FGF21 can also increase insulin sensitivity by promoting the expansion of subcutaneous WAT. *Fgf21* knockout mice show less subcutaneous WAT and a greater degree of insulin resistance. After treatment with recombinant FGF21, subcutaneous adipose tissue was restored with a subsequent improvement in insulin sensitivity (Li et al., 2018). The expression of co-factor beta klotho is necessary to accomplish the FGF21-related expansion of subcutaneous fat (Li et al., 2018). Finally, chronic pharmacologic administration of FGF21 in obese mice has been shown to suppress growth hormone (GH) and the insulin growth factor-1 (IGF1) signaling axis in the liver, increasing lifespan through an improvement in insulin sensitization, normalization of glycemia, and a reduction in body weight (Zhang Y. et al., 2012).

Under normal conditions, FGF21 is synthetized and released from the liver. However, in certain circumstances, such as adaptive thermogenesis induced by cold exposure or exercise, BAT expresses and releasees FGF21 (Chartoumpekis et al., 2011; Hondares et al., 2011; Giralt et al., 2015; Lee et al., 2015). The production of FGF21 by BAT is not negligible; it significantly contributes to systemic FGF21 levels (Hondares et al., 2011). Following exposure to cold, the production of FGF21 by BAT is greater than that of the liver, enhancing thermogenesis, confirming the key roles of BAT in regulating FGF21 levels (Hondares et al., 2011). Moreover, a dramatic rise in Fgf*21* expression in BAT has also been reported in *Ucp1*-null mice or after genetic inactivation of UCP1 protein. In these situations, there is an increase in serum FGF21 levels without changes in FGF21 gene expression in the liver (Keipert et al., 2015; Samms et al., 2015). This suggests thermogenic regulation of FGF21 through both UCP1-dependent as well as UCP1-independent mechanisms (Keipert et al., 2015; Samms et al., 2015).

Taken together, adipose tissue, liver, and skeletal muscle respond to multiple stimuli in order to increase adaptive thermogenesis and induce the browning of WAT (Figure 1). Expression and release of FGF21 by the liver, BAT, and skeletal muscle is induced by shivering (Badman et al., 2007; Inagaki et al., 2008; Hondares et al., 2011), physical activity (Cuevas-Ramos et al., 2010, 2012b; Kim et al., 2013), protein synthesis after growth hormone treatment (Chen et al., 2011), and as a consequence of experimental or clinical mitochondrial dysfunction following DNA mutations (Suomalainen et al., 2011; Keipert et al., 2014). FGF21, together with irisin, insulin, and noradrenaline, provokes metabolically healthy effects that are concomitantly associated with the browning of WAT (Mossenbock et al., 2014; Lee et al., 2015; Villarroya et al., 2017). The higher BAT activity and increased heat production may benefit human health, reducing weight, preventing hyperglycemia, and hyperlipidemia, and protecting against obesity through enhancement of energy waste (Cuevas-Ramos et al., 2012a). These effects explain the association of FGF21 and explain its key role in the “browning” of WAT. This was probably aimed to allow the adaptation of human metabolism to obesity, diabetes, dyslipidemia, metabolic syndrome, and other insulin resistance states (Figure 1).

## FGF21 as Potential Medical Treatment to Induce Browning of WAT

The beneficial metabolic consequences of “browning” may be useful to treat metabolic diseases in humans (Barquissau et al., 2016). There are multiple medical drugs or nutritional inducers that may help to stimulate browning (Table 1). Recently, the role of FGF21 in the browning of WAT has been evaluated with the development of recombinant FGF21-analogs. Initially, clinical trials with analogs LY2405319 and PF05231023 were focused on treating human metabolic diseases including obesity, metabolic syndrome and type 2 diabetes (Gaich et al., 2013; Talukdar et al., 2016). However, resistance to their actions, resulting in an inadequate clinical effect, has been a problem; in humans, only a slight glucose, weight or triglyceride reduction was achieved. The subcutaneous depots of WAT are small cells with greater potential to differentiate (Gustafson and Smith, 2015). Therefore, it is feasible to hypothesize a positive effect if “browning” can be induced, following a sufficient stimulus using FGF21-analogs. In addition, the FGF receptor type 1 together with beta klotho cofactor, called the FGFR1/KLB complex, is the functional target for FGF21 (Kolumam et al., 2015). The use of FGF21 agonist antibodies that specifically activate this complex largely mimic the action of recombinant FGF21 in mice (Kolumam et al., 2015). These include the agonist antibodies BFKB8488 and NGM313, which are currently under clinical research (ClinicalTrials.gov, NCT02593331, NCT02708576, and NCT03060538). Both FGF21 recombinant analogs (Gaich et al., 2013; Talukdar et al., 2016) and the FGFR1/KLB complex agonist antibodies induce higher thermogenesis and browning of BAT through UCP1-dependent pathways. Although UCP1 has traditionally been thought of as indispensable for browning and thermogenesis (Golozoubova et al., 2001; Feldmann et al., 2009), recent research suggests a role of a UCP1-independent pathway (Chen et al., 2017). In *Ucp1* KO mice, higher thermogenesis with weight loss and beneficial changes in cardiometabolic markers have been reported (Veniant et al., 2015; Chen et al., 2017). The origin of this UCP1-independent thermogenesis is still controversial, with different reports suggesting the opposite (Keipert et al., 2015). Further investigation is warranted to clarify if higher thermogenesis can be obtained without overexpression of UCP1.

In addition to FGF21-recombinant analogs or the FGFR1-KLB complex agonist antibodies, other drugs have been used with similar aims; however, most have shown little clinical utility. For example, PPAR-alfa agonists (fibrates), adrenergic beta-3-receptor stimulators, thyroid hormones, and more recently irisin have been tested (Table 1). There are also certain nutritional inducers of “browning” of WAT that may be considered as therapeutic options. The most important hormones, drugs and nutritional inducers of browning are summarized on Table 1. It is important to mention that although nicotine has been associated with body weight reduction, mainly due to the associated decreased appetite, greater lipolysis, and increased energy waste (Zoli and Picciotto, 2012), it has never been confirmed that smoking cigarettes can induce browning (Chen et al., 2005).

## Conclusion

FGF21 is a key regulator of the differentiation of WAT to brown adipocytes, resulting in enhanced thermogenesis and energy waste. The main action seems to be through UCP1-dependent and -independent mechanisms. In addition, after cold exposure or exercising, FGF21-induced upregulation of local peroxisome proliferator-activated receptor gamma co-activator (PGC)-1-alfa increases thermogenesis in adipose tissue and skeletal muscle. Potential mechanisms involve higher noradrenaline levels that act on the WAT-beta-3 adrenergic receptor, inducing lipolysis, and higher serum free fatty acids, which in turn increase PPAR-alfa agonism at liver, and higher FGF21 synthesis and then release into circulation. This effect contributes to other FGF21-related mechanisms that integrate metabolic pathways to regulate human energy balance, glucose and lipid levels. Therefore, the development of better recombinant FGF21 analogs as a potential treatment for metabolic diseases in humans is necessary.

## Author Contributions

All authors made substantial contributions to the conception and/or design of the work; acquisition, analysis, and interpretation of data for the work; drafting the work or revising it critically for important intellectual content; and approved the final version of the article to be published.

## Conflict of Interest Statement

The authors declare that the research was conducted in the absence of any commercial or financial relationships that could be construed as a potential conflict of interest.
